# Racial Bias in Neural Empathic Responses to Pain

**DOI:** 10.1371/journal.pone.0084001

**Published:** 2013-12-23

**Authors:** Luis Sebastian Contreras-Huerta, Katharine S. Baker, Katherine J. Reynolds, Luisa Batalha, Ross Cunnington

**Affiliations:** 1 The University of Queensland, Queensland Brain Institute, St Lucia, Queensland, Australia; 2 Laboratory of Cognitive and Social Neuroscience (LaNCyS), UDP-INECO Foundation Core on Neuroscience (UIFCoN), Universidad Diego Portales, Santiago, Chile; 3 Centre for the Study of Argumentation and Reasoning, Universidad Diego Portales, Santiago, Chile; 4 The University of Queensland, School of Psychology, St Lucia, Queensland, Australia; 5 Department of Anesthesiology, Stanford University School of Medicine, Stanford, California, United States of America; 6 Research School of Psychology, Australian National University, Canberra, Australian Capital Territory, Australia; University of Maryland, College Park, United States of America

## Abstract

Recent studies have shown that perceiving the pain of others activates brain regions in the observer associated with both somatosensory and affective-motivational aspects of pain, principally involving regions of the anterior cingulate and anterior insula cortex. The degree of these empathic neural responses is modulated by racial bias, such that stronger neural activation is elicited by observing pain in people of the same racial group compared with people of another racial group. The aim of the present study was to examine whether a more general social group category, other than race, could similarly modulate neural empathic responses and perhaps account for the apparent racial bias reported in previous studies. Using a minimal group paradigm, we assigned participants to one of two mixed-race teams. We use the term race to refer to the Chinese or Caucasian appearance of faces and whether the ethnic group represented was the same or different from the appearance of the participant' own face. Using fMRI, we measured neural empathic responses as participants observed members of their own group or other group, and members of their own race or other race, receiving either painful or non-painful touch. Participants showed clear group biases, with no significant effect of race, on behavioral measures of implicit (affective priming) and explicit group identification. Neural responses to observed pain in the anterior cingulate cortex, insula cortex, and somatosensory areas showed significantly greater activation when observing pain in own-race compared with other-race individuals, with no significant effect of minimal groups. These results suggest that racial bias in neural empathic responses is not influenced by minimal forms of group categorization, despite the clear association participants showed with in-group more than out-group members. We suggest that race may be an automatic and unconscious mechanism that drives the initial neural responses to observed pain in others.

## Introduction

Empathy is defined as the ability to comprehend and vicariously share the feelings and thoughts of other people, according to the perception-action model [1]. These feelings have an evolutionary role fomenting altruistic behaviours [2] and act as a key motivator in help and co-operation [3]. They are the proximate mechanism by which an individual perceives and shares in the distress of another person [4]. Hence, empathy may have an evolutionary origin as a mechanism selected to foment altruistic behaviours in human societies toward a common welfare [2].

Recent studies have shed light on the neural mechanisms that underlie empathic feelings, in particular empathy elicited by the perception of pain in others. Imaging studies have shown that when people see or imagine the pain of another person, they map that observed pain onto their own brain network which is activated during firsthand experience, as if they were vicariously experiencing the pain (e.g. [4]–[10]). The areas typically showing an empathic neural response to observed pain include somatosensory cortex (e.g. [6], [11]–[15]), and areas involved in the motivational-affective dimension in the pain matrix such as bilateral anterior insula (AI) and anterior cingulate cortex (ACC) (e.g. [4], [11], [13], [16]–[22]). Thus, there is a “shared representation” between the self and the other in pain that may be the basis of the affective empathic feelings.

Nevertheless, there is converging evidence to suggest that empathy in humans is more complex than a mere resonance with the target's painful state. Indeed, cognitive and affective factors can modulate the activation of neural patterns in empathy [7], [17], [22], [23]. Furthermore, recent imaging studies have found that social and contextual factors can also regulate empathic neural responses to others' pain [10], [11], [24]–[28], including race of the target person [29]–[34]. This racial bias has been seen not just in empathy for pain, but also in empathic responses to facial emotions [33], [35]. Such studies build on evidence that racial bias is a potent modulator of neural responses underlying many social behaviours [36]–[41].

In a recent study, reduced neural empathic activity was found when participants viewed people of another race receiving a painful touch compared with people of the same race [34]. In that study, Caucasian and Chinese participants were scanned while they watched video clips of Caucasian or Chinese actors, with a neutral facial expression, receiving either painful or non-painful touch on their cheek (with a syringe needle or cotton-bud respectively). Notably, empathic pain activity in the anterior cingulate cortex and left insula was significantly less when participants viewed painful touch to the faces of other-race compared with same-race people.

This finding has been supported by other recent studies that also report racial biases in neural empathic responses. For instance, activation in the anterior insula cortex [30] and muscle-specific cortico-spinal inhibition measured by transcranial magnetic stimulation (TMS) [29] are both greater in response to pictures of hands in painful situations when those hands are from people of the same race as the participant compared with a different race. Furthermore, this effect is correlated with implicit measures of racial bias [29]. Similarly, in studies of autonomic arousal, reduced skin conductance responses have been shown when participants observe pain in other-race people compared with same-race people [31]. Finally, greater activation within the medial prefrontal cortex, an area associated with more cognitive aspects of empathy, has been shown in response to naturalistic visual scenes depicting emotional suffering of own race relative to other race people, and the level of this racial bias in neural empathy also predicted greater altruistic motivation for same-race members [32].

It has therefore been suggested that, relative to cultural influences, the modulation of empathy by racial group membership is more fundamental and plays a more pivotal role in shaping social behaviours, perhaps due to an evolutionary history of coalitions and alliances between ethnic groups [31], [34], [42]. The underlying cause of racial bias in neural empathic responses is, however, still unknown.

Racial bias in empathic responses may stem from a more broad or general in-group/out-group bias, rather than being caused by race per se. Race can help people to define themselves as part of a specific group, eliciting empathic feelings towards in-group partners. Thus, activity in the affective areas, associated with empathic neural responses, may mediate emotions and feelings shared by the in-group, not implicating explicit consciousness of these feelings. Indeed, affective feelings towards an in-group member have been found to increase resource sharing and helping behaviour among diverse social groups [43] such as political affiliations [44], sport team allegiances [45], as well as race [46]. In fact, a recent functional magnetic resonance imaging (fMRI) study demonstrated an in-group modulation in neural empathic responses to pain, showing stronger brain activation in the left anterior insula cortex when participants witnessed pain of an in-group member (a fan of the same football team) as compared with an out-group member (a rival team), and this effect was associated with greater frequency of helping behaviour [47]. This same pattern of activation in anterior insula has also been found in response to negative experiences of people belonging to the participant's own group compared with those in the rival team [48]. These studies confirm that similar effects as found in race modulation of neural empathy are also shown in other forms of social group categorisation.

The way in which racial bias and broader social group biases may be related in neural empathic responses has rarely been examined. Racial bias, however, can be modulated by a more general in-group bias in other cognitive tasks. Van Bavel and collaborators have shown this relationship in both behavioural and neural responses [49]–[51]. In their studies, participants who were randomly assigned to a mixed-race (White and Black) group, using a minimal group paradigm, developed more positive evaluations of in-group members compared with out-group members in implicit measures, without any effect of race [49]. Moreover, greater activation was found in areas associated with face recognition when observing members of the in-group compared with out-group, regardless of race [50], [51]. While previous studies have shown racial bias in both implicit tasks and face recognition [37], [52], results of these studies of minimal (mixed-race) groups suggest that mere categorization with a relatively arbitrary group may be sufficient to override automatic evaluations and biases relating to race.

Likewise, the artificial division of people into two groups by a minimal group paradigm can be sufficient to facilitate in-group bias in empathy for pain [53]. In a recent study, participants were shown pictures of painful and non-painful situations and were asked to judge the level of pain when imagining either themselves, an in-group member, or an out-group member in that situation. Pain ratings were significantly greater when participants viewed pain from an in-group member perspective, suggesting greater empathic feelings toward in-group members, even when social group categorisation was non-relevant and arbitrary [53].

Only one recent study has examined relationships between race and minimal-group biases in neural empathy for pain, using electroencephalography (EEG) [33]. In this study, Chinese participants viewed pictures of Chinese and Caucasian actors' faces with a painful or neutral facial expression. Participants showed greater activity in early face-related processing components of the event-related potentials (ERPs - P2 and N2), but only in response to Chinese faces. However, when participants were randomly assigned to one of two mixed-race minimal-groups, the racial biases in face-processing components were abolished for in-group faces although still present for out-group faces. Therefore, mere categorization as an in-group or out-group member can modulate racial biases seen in the neural processing of facial emotion. However, as the neural sources of activity are difficult to localise from EEG studies, it is still not known how racial biases seen in brain regions important for affective aspects of empathy may be related to broader group categorisation.

The aim of this study was therefore to examine whether a more general in-group membership categorization could explain or modulate the racial bias typically seen in neural empathic responses to observed pain. We recruited Caucasian Australian participants and divided them randomly into one of two mixed-race groups (Caucasian and Chinese). Participants were first shown pictures and learnt to remember the members of their “own group” and the “other group”. Then, during fMRI measurement, participants were shown brief videos of the faces of their in-group and out-group members either receiving a painful or a non-painful touch on the cheek, following the experimental design of Xu et al [34]. Participants also completed an affective priming task, using pictures of faces of their in-group and out-group members as primes, in order to test for implicit association with their group. We were therefore able to examine whether neural responses to observed pain, in affective processing regions of the brain, would be more greatly influenced by the race of the observed person or by their social categorization as in-group or out-group members regardless of race.

## Materials and Methods

### Participants

Twenty Caucasian-Australian participants (8 males; mean age = 22.5, SE = 1.06 years, 2 left-handers) were recruited through the University of Queensland, and received AU$30 as reimbursement. The criteria to consider participants as Caucasian-Australian were being born in Australia, having white skin, and having Caucasian, Anglo-Saxon parents. All had normal or corrected-to-normal vision and reported no abnormal neurological or psychiatric history.

### Ethics Statement

All participants gave written consent to take part in the study (as outlined in PLOS consent form) in agreement with the Helsinki declaration. This study was approved by the Medical Research Ethics Committee of the University of Queensland. All data were analyzed anonymously.

### Procedure

Each participant attended two experimental sessions in which they were first assigned to a group and photographed (session 1). They then undertook fMRI measurement while observing video clips of painful versus non-painful touch and performed an affective priming task to test implicit group association (session 2).

### Group Assignment

Participants were informed that they were taking part in a study focusing in the neural responses to other people's emotion, and that they would be divided into two groups in order to compare their brain responses when watching emotions of members of their group compared with members of another group. We were very careful to never reveal to participants that the race of in-group and out-group members was a factor, until after completion of the study. For group assignment, participants completed a 10-item questionnaire assessing authoritarian and moral attitudes [54]. We explicitly told participants that they would be assigned to a group with people who shared the most similar beliefs and attitudes to them, and that the other group rated most differently on their beliefs and attitudes. In reality, group assignment was random. Participants were also photographed so that their photo could be included with the members of their group during the subsequent team learning task.

### Team Learning Task

The second session took place between 3 to 5 days after session 1. In this session, participants first completed a learning task that took approximately 10 minutes. They were told that they would be shown photos of members of their group and the other group, and that they should learn and recognize each person so they could identify who belonged to their group and who belonged to the other group. Crucially, in each group, there were 2 Caucasian (1 male, 1 female) and 2 Chinese (1 male, 1 female) actors, making 8 actors in total with own-group/other-group, Caucasian/Chinese, and male/female balanced in a 2×2×2 design. The same photographs of the 8 actors were used for all participants, but the actors assigned as own-group or other-group were pseudo-randomised and counter-balanced between participants so there could be no overall group bias introduced by the photograph of any particular actor(s) in the set.

In the initial learning phase, three blocks of trials were displayed in which the photos of members of the participant's own team (including the participant) and the other team were shown sequentially (stimulus duration 2 s; inter-trial interval 3 s). Below each photo, the text “Your Group” or “Other Group” was displayed so that participants could learn the group to which each actor belonged. The photos of the 8 actors, plus the photo of the participant, were shown sequentially 3 times each, for a total of 27 trials.

Participants' recognition performance for the faces was then tested. Photos of the 8 actors, plus the participant's own photo, were presented in random order with no text identifying groups. Participants reported verbally the group to which the face belonged by saying “My group” or “Other group”. Verbal report was used rather than button-responses to avoid participants learning any association between own/other group and left/right responses which would cause confounds for the later affective priming task (see below). The participant's verbal responses were coded by the experimenter and feedback was given by text displayed beneath the photo: “Your Group” or “Other Group” displayed in red-font for incorrect responses and in green font for correct responses. The photos were presented in blocks of 9 trials (each face presented once in random order), and blocks were repeated until participants met the criteria for recognition performance: 4 consecutive blocks (36 trials) performed with less than 3 errors in total (>90% correct) and 100% correct in the last block. Participants performed a further 4 test blocks inside the scanner immediately prior the fMRI task, to ensure that they still accurately recognized the faces as own-team and other-team immediately before beginning the fMRI task.

### fMRI Task

**Figure 1:**
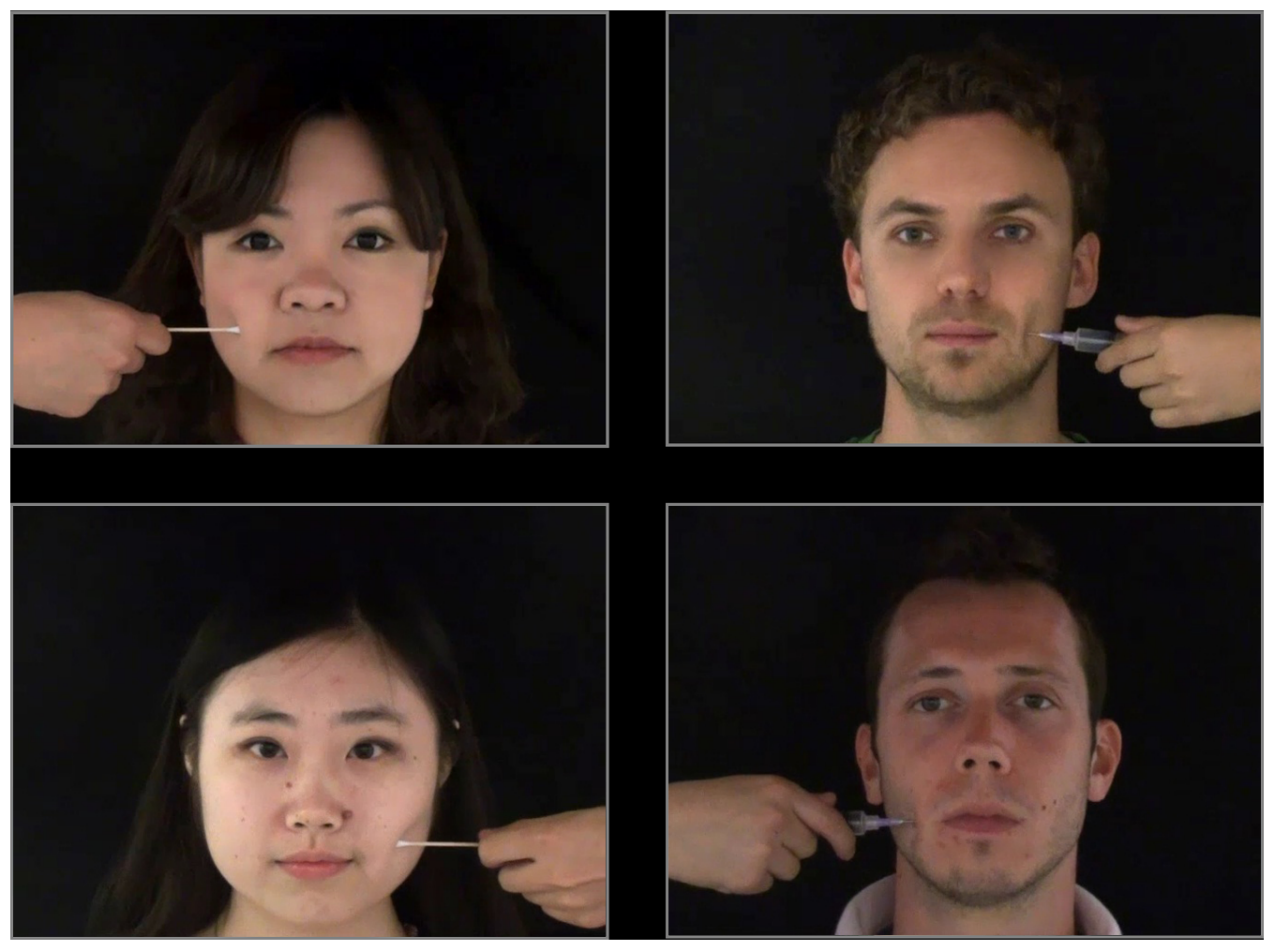
Stimuli used during the fMRI task. Participants watched video clips depicting Asian or Caucasian actors who represented members of own-group or other-group receiving either painful (syringe needle) or non-painful (cotton-bud) touch on the left or right cheek. Actors included in this figure have given written informed consent for publication of their photograph, as outlined in the PLOS consent form.

Inside the scanner, during fMRI measurement, participants watched short video clips of each of the actors being touched on the cheek either by a cotton-tip (non-painful touch) or by a syringe needle (painful touch). The task followed the design of the study of Xu et al [34]. The stimuli consisted of 32 video clips, each of 3 s duration, showing faces of the 8 actors with a neutral facial expression receiving either a painful touch (syringe needle) or non-painful touch (cotton-tip) to either their right or left cheek (4 video clips per actor). Importantly, the video clips portrayed only the cotton-tip or syringe-needle, held by a hand with identical grip, moving towards the cheek of the actor and ended immediately upon contact of the cotton-tip or needle with the actor's cheek, so that no facial expression of pain or emotional responses of the actors to the touch were portrayed (Fig. 1). Video clips were displayed on a projection screen in the bore of the scanner at a viewing distance of 80 cm, and were viewed by participants via a mirror attached to the head coil of the scanner. Following each video clip, participants were instructed to rate how painful they thought the stimulus looked by pressing one of four buttons on a button-box held in their right hand, rating from no pain (left button) to considerable pain (right button). Each of the 32 video clips were presented once in random order, with each 3 s duration video clip followed by 9 s of fixation-cross (12 s fixation before the first video), for a total fMRI run duration of 6 min 40 s. Participants completed 4 fMRI runs, with the 32 video clips presented in a different random order in each run.

### Implicit Team Association: Affective Priming task

Following fMRI measurement, outside the scanner, participants performed an affective priming task in order to test for implicit group association. In this task, the photos of the 8 actors, as used in the learning and recognition tests, were presented as primes and paired with words of positive or negative valence. The design of this task was based on similar previous studies [49], [55]. Each trial started with a blank screen (1000 ms) followed by a fixation cross (500 ms) as a warning cue. The prime photo was presented centrally (200 ms), followed by a blank screen (100 ms), and then a target word of either positive valence (‘charming’, ‘nice’, ‘friendly’, ‘happy’, ‘desirable’, ‘kind’) or negative valence (‘repulsive’, ‘nasty’, ‘evil’, ‘angry’, ‘disturbing’, ‘annoying’) was presented until a response was made (with 3000 ms maximum time-out). On presentation of the target word, participants responded by button-press to classify the word as pleasant or unpleasant as quickly as possible, pressing the corresponding button on a 2-button response box. Reaction times to correctly classify the words as pleasant or unpleasant were measured as the dependent variable. The mapping of left/right responses to pleasant/unpleasant was counterbalanced between participants. After classifying the word by button-press, participants verbally reported whether the prime face was a member of their own group or the other group. This ensured that participants' attention was drawn to the group membership of the face when the prime photo was presented. Verbal report was used for classifying faces as own/other group so that, as with the earlier recognition test, participants did not learn any association between left/right responses and own/other team that might have interfered with button-press responses to the words in the affective priming task. Participants performed 96 trials in randomized order, consisting of each of the 8 photos of actors paired once with each of the 6 pleasant and 6 unpleasant words. Reaction times and accuracy (% correct) to classify words as pleasant or unpleasant were analyzed by 3-way ANOVA with factors of Race (Caucasian/Chinese), Group (in-group/out-group) and Valence (pleasant/unpleasant). Mauchly's test of sphericity was checked and Greenhouse-Geisser corrections for non-sphericity applied where appropriate. Furthermore, we performed Kolmogorov-Smirnov goodness-of-fit tests over the residuals of the dependent variables in order to check that the distribution of these data did not deviate significantly from a normal distribution.

### Explicit Team Identification

Finally, participants completed a short questionnaire to assess the degree to which they explicitly identified with their group and the other group. Three questions were given: ‘How similar do you see yourself to be to members of your team?’; ‘How similar do you see yourself to be to members of the other team?’; ‘To what extent do you see members of your team and the other team to be similar to each other?’. Participants answered questions using a 5-point Likert scale from 1, not at all similar, to 5, very similar.

### fMRI image acquisition and analysis

The fMRI data were collected on a 3 Tesla Siemens MRI scanner. Functional images were acquired using gradient-echo echo-planar imaging (EPI) with the following parameters: 38 horizontal slices (3 mm slice thickness + 10% inter-slice gap, interleaved acquisition), repetition time (TR) = 2.5 s; echo time (TE) = 35 ms; field of view (FOV) = 190 mm; flip angle (FA) = 90°; matrix of 64×64 voxels at 3 mm^2^ in-plane resolution. 159 brain volumes were acquired in a run duration of 6 min 40 s, with a total of 636 volumes acquired over 4 fMRI runs. The first 5 images of each run were discarded as dummy scans to allow the MR signal to reach a steady-state. Anatomical T1-weighted images were also obtained covering the entire brain (TR = 1900 ms, TE = 2.3 ms, FA = 9°, matrix = 256×256 voxels, slice thickness = 0.9 mm).

Data preprocessing and analysis was performed using SPM8 software (Wellcome Trust Centre for Neuroimaging, London, UK). Slice timing correction was first applied to correct for the acquisition time differences between slices during the sequential imaging. The functional images were then spatially realigned to the first image to correct for head motion between scans. The anatomical T1 image was first coregistered to the mean functional image and then spatially normalized to the standard MNI T1 template using the Segment routine of SPM8. This same registration to MNI space was then applied to all functional images. Finally, functional images were resliced to 2×2×2 mm resolution and spatially smoothed using a Gaussian filter of 6 mm full-width/half-maximum (FWHM). For data analysis, event-related neural activity was modeled at the onsets of each of the 8 types of videos, convolved with the canonical hemodynamic response function. The eight conditions modeled were Painful and Nonpainful touch for each of the 4 types of faces shown: Caucasian/in-group, Caucasian/out-group, Chinese/in-group and Chinese/out-group. A set of 5 contrasts were calculated, comparing Painful versus Nonpainful touch for each of the four face types separately and Painful versus Nonpainful touch averaged across all four face types.

For group statistical analysis, whole-brain SPM analysis was performed using a single-sample t-test to examine the contrast of painful versus non-painful touch averaged across all faces, using a voxel-level probability threshold of P_FWE_<0.005 corrected for multiple comparisons and a cluster extent threshold of 20 voxels. This analysis revealed those regions associated with empathy for pain, showing significantly greater activation when observing painful compared with nonpainful touch averaged across all face types. This empathy for pain network was defined as a mask and used to restrict all subsequent analyses only to voxels within these regions. In this way, all subsequent analyses of race and group effects on empathy for pain were conducted only in those brain regions that showed a significant neural empathic response when averaged across all faces.

For comparisons between face types, the contrasts of painful versus nonpainful touch for each of the four types of faces (Caucasian/in-group, Caucasian/out-group, Chinese/in-group and Chinese/out-group) were entered into a factorial model and analysed by 2-way ANOVA, inclusively masked by the contrast of painful versus non-painful touch averaged across all faces (as above). Main effects of Race (Caucasian versus Chinese) and Group (in-group versus out-group) on empathy for pain activation were examined using a cluster-level probability threshold of P_FWE_<0.05, with clusters defined by the voxel-level threshold P_uncorrected_<0.001. In those areas showing significant activation differences across group or race, individual contrast parameter estimates (i.e. levels of activation) were extracted from the peak voxels and plotted to show neural activation levels between the conditions.

## Results

### Behavioral Data

#### Team Learning Task

During the test blocks, participants on average reached 98% accuracy in categorizing the faces as members of their group or the other group (SE =  0.66%), reaching recognition performance criterion on average in 5.25 blocks (SE =  0.54). In the four test blocks performed inside the MRI, immediately prior scanning, almost all participants performed with 100% accuracy; only three participants made one error each during the four blocks (97% accuracy). Participants were therefore highly accurate in identifying the actors as members of their group or the other group.

#### fMRI task

While observing the video clips, participants rated how painful each stimulus looked on a 4-point scale. Videos showing painful touch were rated as significantly more painful (M =  3.16; SE =  0.08) than videos showing non-painful touch overall (M =  1.10; SE =  0.02; Wilcoxon signed rank test, z =  -7.77, p<0.001). In order to test whether race and/or group membership had an effect on perceived pain ratings, the ratings given for each of the four face types were analysed separately for painful and nonpainful touch with Friedman's tests. For painful touch, participants ratings were found to differ significantly between the faces (X(3) =  9.75, p<0.03), but no significant differences between faces were found for non-painful touch. For painful touch, the differences in pain ratings between the faces were further examined by paired comparisons using Wilcoxon signed ranks tests. These revealed no significant differences but trends towards higher pain ratings for Caucasian faces compared with Chinese faces averaged across group (z = −1.63, p = 0.102), and for in-group faces compared with out-group faces averaged across race (z = −1.77, p = 0.077).

#### Affective Priming task

**Figure 2:**
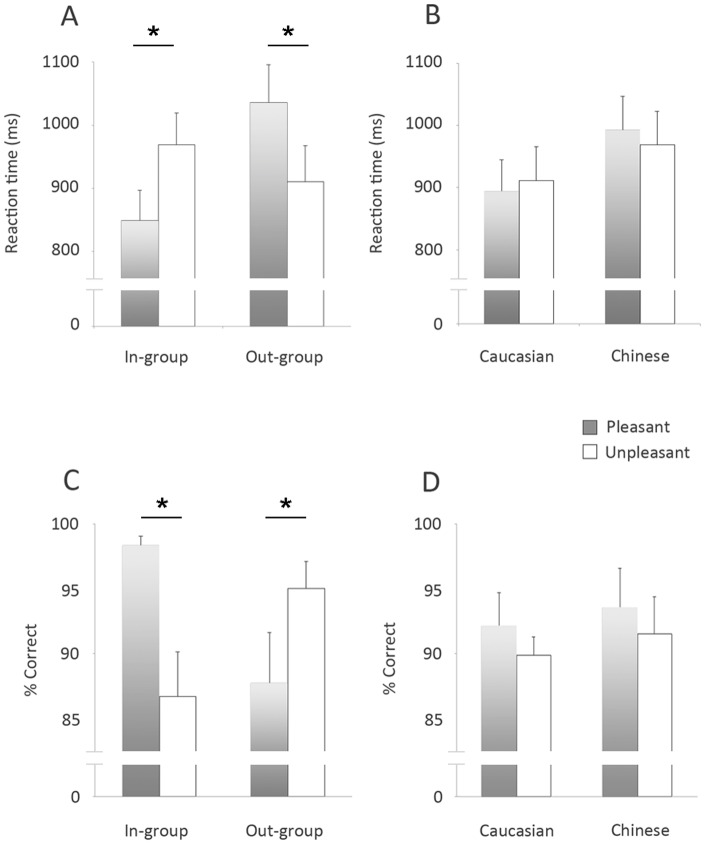
Mean reaction times (top) and % Correct (bottom) in the Affective Priming Task. All 20 participants performed 96 trials on the Affective Priming Task, in which they made button-press responses to classify words as “pleasant” (grey bars) or “unpleasant” (white bars) when primed by faces of each of the 8 actors: either In-group or Out-group members (Left; A and C) and either Caucasian or Chinese faces (Right; B and D). Participants were significantly faster and more accurate classifying words as “pleasant” than “unpleasant” when primed by faces of In-Group members. Conversely, participants were significantly faster and more accurate classifying words as “unpleasant” than “pleasant” when primed by Out-group members. There were no significant differences in response times or accuracy classifying words primed by Caucasian versus Chinese faces.

Reaction times to classify pleasant/unpleasant words in the affective priming task (Figure 2A, 2B) were analyzed by 3-way ANOVA with factors of Race (Caucasian/Chinese), Group (in-group/out-group) and Valence (pleasant/unpleasant), with Greenhouse-Geisser correction for non-sphericity. Kolmogorov-Smirnov tests of goodness-of-fit indicated that these data were normally distributed, and so data were not transformed prior to analysis. ANOVA revealed a significant interaction between group and valence (F(1,19) = 35.97, p<0.001; Fig. 2A), but not between race and valence (F(1,19) = 1.37, p = 0.26; Fig. 2B) and no significant 3-way interaction. Post-hoc analyses revealed that, when primed by faces of in-group members, reaction times were significantly shorter to pleasant words (M =  849 ms, SE =  48 ms) than to unpleasant words (M =  969 ms, SE =  50 ms; F(1,19) =  24.47, p<0.001), whereas when primed by faces of out-group members, reactions times were significantly longer to pleasant words (M = 1036 ms, SE =  60 ms) than to unpleasant words (M =  910 ms, SE =  57 ms; F(1,19) =  15.37, p<0.001).

Participants' accuracy in classifying pleasant/unpleasant words (Figure 2C, 2D) was also analysed by 3-way ANOVA (with Greenhouse-Geisser correction) with the same factors. Kolmogorov-Smirnov tests of goodness-of-fit indicated that these data were normally distributed, and so data were not transformed prior to analysis. The 3-way ANOVA revealed a significant interaction between Group and Valence (F(1,19) = 13.403, p<0.002; Fig.2C), but not between Race and Valence (F(1,19) = 0,012, p = 0.913). Post-hoc analysis revealed identical effects to those found for reaction times: for in-group face primes, percentage accuracy was significantly higher classifying pleasant words (M =  98%, SE =  0.7%) than unpleasant words (M =  87%, SE =  3.9%; F(1,19) =  8.89, p<0.008), whereas for out-group face primes, accuracy was significantly poorer classifying pleasant words (M =  87.71%, SE =  3.44%) than unpleasant words (M =  95%, SE =  2.1%, F(1, 19) =  4.19, p<0.05). Taken together, these results indicate participants associated “pleasant” with in-group faces, showing faster and more accurate responses to pleasant words when primed by in-group faces, and associated “unpleasant” with out-group faces, showing faster and more accurate responses to unpleasant words when primed by out-group faces.

### Explicit Team Identification

Participants gave significantly higher ratings for how similar they judged themselves to be relative to members of their own team (M =  3.05, SE =  0.2) than members of the other group (M =  2.50, SE =  0.18), Wilcoxon signed rank test, z = −2.81, p<0.005.

Overall, these results show that the minimal group paradigm used in this study did result in participants associating more with their in-group than the out-group, as assessed by both implicit and explicit measures of group identification.

### fMRI data

**Figure 3:**
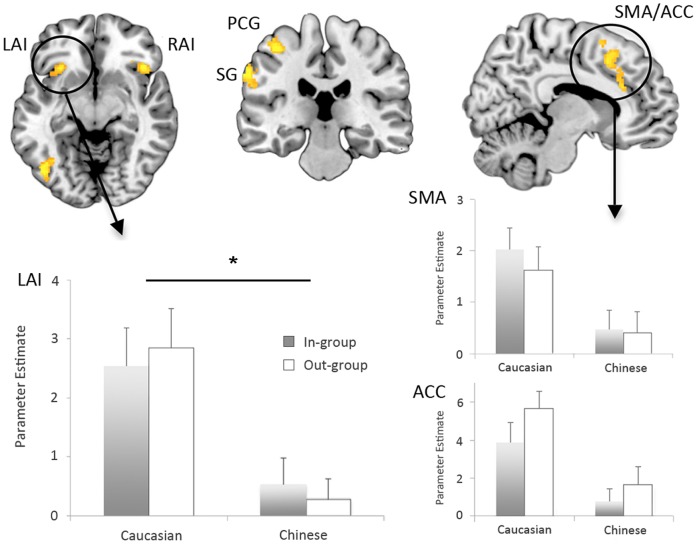
Activation results in the fMRI task. Significantly greater activitation when observing painful versus non-painful touch was found in the left anterior insula (LAI), right anterior insula (RAI), postcentral gyrus (PCG), supramarginal gyrus (SG), and in the supplementary motor area and anterior cingulate (SMA/ACC). Significant differences when viewing painful touch in Caucasian versus Chinese faces were found only in the left anterior insula, with no differences between In-Group versus Out-Group faces (cluster-level P_FWE_<0.05; parameter estimates plotted below left). Similar effects in the SMA/ACC failed to reach significance but are shown here for comparison (at voxel-level P_uncorrected_<0.001; parameter estimates below right).

Observing painful compared with non-painful touch, averaged across all faces, involved significantly greater activation in regions of the supplementary motor area (SMA), mid cingulate cortex and anterior cingulate cortex (ACC), as well as activation bilaterally in the anterior insula (AI) (Fig. 3). Significantly greater activation was also found in primary and secondary somatosensory areas, involving the postcentral gyrus, supramarginal gyrus, and inferior parietal cortex (IPC) (Fig. 3). These included areas 1, 2, 3a, 3b, OP1 and OP4, and parietal operculum as defined by cytoarchitectonic probability maps from the SPM Anatomy Toolbox [56]. Finally, greater activation for painful touch was also found in right cerebellar areas, the left inferior frontal gyrus, and left inferior occipital gyrus (see Table 1).

**Table 1:** Brain regions showing significantly greater activation for painful touch compared with non-painful touch, averaged across all faces.

	MNI coordinates				
Region	x	y	z	t value	Cluster size (N° of voxels)
R Anterior Insula	36	28	2	7.55	274
L Anterior Insula	−28	22	−12	6.68	191
SMA and MCC	−8	20	42	7.03	660
ACC	−10	30	26	5.80	36
Postcentral Gyrus	−38	−24	56	6.85	75
Supramarginal Gyrus	−60	−22	28	6.90	194
Inferior Parietal Cortex	−32	−52	46	6.24	102
Inferior Frontal Gyrus	−56	10	10	5.26	21
Inferior Occipital Gyrus	−42	−66	−10	7.06	234
Cerebellum	36	−60	−28	5.78	95

To examine differences in empathic neural responses to pain between the four types of faces, contrasts comparing Group (own-group versus other-group) and Race (Caucasian versus Chinese) were examined, inclusively masked by the contrast above (i.e. in regions that showed significant neural empathic responses averaged across all faces). For Group comparisons, there were no areas that showed significantly greater activation when observing painful versus non-painful touch in own-group members compared with other-group members, or vice-versa, even if using a more lenient uncorrected threshold of *P*<0.001. Thus, group membership of the observed actor did not significantly modulate empathic neural responses to observed painful touch. However, regarding Race, significantly greater activity was found in the left anterior insula cortex when participants observed painful versus non-painful touch in actors of the same race compared with actors of the other race (cluster-P_FWE_<0.05, peak T = 4.08, MNI coordinates: −28, 24, 8; Fig. 3). The left AI was the only area to show this racial bias effect at the corrected probability threshold; however, a number of other areas showed similar activation differences at the same voxel-level threshold (P_uncorrected_<0.001), but with smaller clusters not reaching the corrected cluster-level statistical threshold. These regions (Fig. 3) included the IPC (peak T = 3.73, MNI coordinates: −34, −34, 38), the left postcentral gyrus (peak T = 3.73, MNI coordinates: −38, −24, 58), the SMA (peak T = 3.28, MNI coordinates: = −6, 14, 58), and the ACC (peak T = 3.21, MNI coordinates = 12, 24, 28). There were no areas that showed significantly greater activation for painful versus non-painful touch in other-race faces compared with own-race faces, even at the more lenient uncorrected threshold, P<0.001.

In order to further test for possible sub-threshold differences in neural empathy to in-group versus out-group members, we extracted parameter estimates from the peak voxels in those regions showing racial bias in neural empathy and further analysed by 2-way ANOVA (effectively using an uncorrected threshold P<0.05 in those selected regions of interest). Results revealed no main effects of Group in any of those areas (left AI, F(1,19) = 0.007, p = 0.934; ACC, F(1,19) = 2.964, p = 0.101; SMA, F(1,19) = 0.309, p = 0.585; IPC, F(1,19) = 0.021, p = 0.886 and postcentral gyrus, F(1,19) = 1.573, p = 0.225), and also no significant interactions between Race and Group in any of those areas (left AI, F(1,19) = 0.208, p = 0.654; ACC, F(1,19) = 0.023, p = 0.881; SMA, F(1,19) = 0.122, p = 0.73; IPC, F(1,19) = 0.619, p = 0.441 and postcentral gyrus, F(1,19) = 0.244, p = 0.627). We found only significant main effects of Race in all areas, consistent with the whole-brain analysis.

## Discussion

Overall, when participants witnessed others receiving painful versus non-painful touch, we found enhanced activation in the core neural network for pain empathy, including somatosensory and affective-motivational aspects of pain processing, consistent with previous results in empathy-for-pain research [57]. Crucially, while group assignment clearly led to greater association with in-group rather than out-group members in both explicit and implicit measures, we found no significant group bias in the neural response to observed pain. Instead, neural empathic responses showed only a significant race bias, regardless of group, with activation in the left insula cortex significantly greater when observing painful touch in same-race compared with other-race actors, consistent with previous studies [34]. Other regions typically reported as part of the neural empathy for pain matrix, including the anterior cingulate and left somatosensory areas, also showed a similar trend towards racial bias (although the size of these clusters was not large enough to reach our strict cluster-corrected threshold for statistical significance).

Race has been demonstrated as a feature impossible to ignore in facial processing [38], [58]–[60], even when race is implicit and not relevant to the participant's task. Thus, it is possible that race may cause an automatic and bottom-up bias in empathic neural activation to pain. It may be that the neural processing for differentiation of race operates at a more basic level than broader social distinctions. Previous ERP studies have shown that racial information is extracted and encoded from faces at a very early stage of facial processing, as early as 120 ms in the N100 component of the ERP, before the analysis of more complex social categories [58]–[60]. Hence, this automatic and rapid encoding of race in the brain may be underlying the current results.

It is possible that early effects of race in facial processing might be due to differing physical features of faces according to race, such as color, shape and size [61]. The anterior insula and anterior cingulate cortex that show a racial bias in neural empathy are also known to be sensitive to salient physical features of a stimulus and bottom-up information processing [62]. In contrast, group membership in our paradigm was not associated with any salient physical features that might elicit early and automatic responses that could modulate effects of race. Thus, in our paradigm, the more complex social categorisation of group valence could only be detected in later stages of processing after the recognition of facial identity and matching with remembered in-group and out-group members. Therefore, it is possible that earlier neural processing of race based on physical features rather than other complex social categories as in our minimal-group paradigm may regulate the neural empathic responses to observed pain.

In the study by Sheng and Han [33], empathic neural activity was modulated by both race and group membership when salient physical cues (different color t-shirts) were used to identify in-group and out-group members. Such salient physical cues may facilitate an automatic and early identification of in-group members that could compete with low-level visual features of faces corresponding to race to influence neural empathic responses. While in our study participants were highly accurate in recognizing individuals as in-group and out-group members, based on facial identity, providing low-level cues for group membership may be necessary for such arbitrary group categorization to influence early neural processes and modulate the racial bias in empathy for pain. Han et al., [28] also found that observing neutral faces in painful situations elicited stronger empathic neural activation than observing painful facial expressions in the same situation (being touched by a syringe on the cheek, similar to our task). It may be that this strong activation to painful situations, without concurrent processing of emotional facial expressions, is less influenced by group manipulation. In the study of Sheng and Han [33], painful and neutral facial expressions were used to represent painful and non-painful conditions. Previous studies have shown that processing of facial expressions, with more complex characteristics than neutral faces, recruits areas involved in mentalizing and theory of mind such as the medial prefrontal cortex and inferior frontal gyrus [63], [64]. Perception of nociceptive touch, however, may stem more directly from sensori-motor activity, perhaps involving “mirroring” mechanisms [65], that may be less influenced by higher-order social group categorization.

Since the anterior insula cortex is involved in the integration and representation of interoceptive and affective information and the anterior cingulate cortex is its motivational and action empathic counterpart [66], [67], race bias in these areas suggests a decrease in affective-automatic response to pain in other-race faces. This is consistent with previous studies that have shown the involvement of the anterior cingulate and anterior insula in emotional face processing (for a review see [68], [69] ), as well as in racial discrimination in its affective and cognitive processing (e.g.[70]–[72]). Likewise, brain areas that are densely connected with the insula such as the amygdala and parahippocampal cortex [73]–[76], show differing activation in response to same versus other-race faces [37], [77]–[79], suggesting differentiation in the processing of races in both emotional and cognitive aspects. Furthermore, ERP studies have shown that racial bias in neural empathic responses to painful facial expressions occurs early in processing, around 200 ms after stimulus onset over frontal areas and localized to the anterior cingulate [33]. Taken together, this would imply an automatic and bottom-up bias in affective processing of empathy driven by the race of a face.

Besides race, there are many other factors that can also modulate empathic responses to pain, involving complex mechanisms such as contextual appraisal or evaluation of intentions that are associated with the cognitive dimension of empathy [9], [11], [13]. Indeed, studies that have investigated the modulation of neural empathic activity based on culture and more complex social constructs have reported additional recruitment of cognitive areas, reflecting a more complex and top-down regulation of empathy [32], [80]. A recent study of intentional empathy showed no racial bias in neural empathic responses to facial emotions, but additional recruitment of inferior frontal regions independent of race when intentionally empathizing with the observed emotional state [81]. This suggests that cognitive aspects of empathy may involve engagement of more prefrontal cortical regions and regulate the more automatic emotional empathic responses that appear to be sensitive to race.

It is also possible that modulation of racial bias in the affective brain regions requires more meaningful or established social categorisation than the minimal group paradigm. For example, a recent study by Bruneau et al. [82] examined neural activity of Arabian, Israeli, and South American participants in response to the pain and suffering of people from each group. Their results showed that, behaviorally, there was a reduction in the participants' compassion towards the conflict group (i.e. Arab to Israeli and vice-versa), but not for the distant group (Arab and Israeli to South American and vice-versa). At the neural level, fMRI showed greater activation in areas associated with cognitive aspects of empathy, such as the medial prefrontal cortex, in response to the conflict group but not to the distant group. These results show how more complex group categorization, rather than simple minimal groups or race alone, can modulate cognitive aspects of empathy and perhaps exert top-down control over empathic responses to observed pain of others. Empathy has been proposed as an evolutionary mechanism to facilitate pro-social motivation and behavior toward conspecifics [83], [84], and it is widely accepted that social group membership is involved in the elicitation of empathic feelings and altruism [43]–[47]. However, it seems that artificial and arbitrary groups, as in a minimal group paradigm, are not sufficient to override racial bias in empathy. It should be noted that our group assignment was not strictly “minimal” as we also told participants that they shared similar beliefs and attitudes as others in their group; however, even so this was not sufficient to influence the neural responses to observed pain.

In our study, we found significant group biases when measured on implicit and explicit tasks assessing group association behaviourally. These results are important to show that our group assignment was effective in inducing greater association or identification with in-group members than out-group members. While these behavioural results may at first seem at odds with the fMRI activation results, in which no group biases were found, the affective priming task and explicit ratings were not measures of empathy towards in-group versus out-group members. Therefore, while our affective priming task showed that participants implicitly associated positively with in-group members and negatively with out-group members in general, this group association did not influence the neural empathic responses shown by participants to observed pain of in-group compared with out-group members. The pain ratings in the fMRI task showed no significant differences, but trends towards higher ratings of pain to faces of same-race compared with other-race individuals, as well as in-group compared with out-group members. These pain ratings are the most equivalent behavioral measure to the neural empathic responses measured in the fMRI task. Unfortunately these measures were not very sensitive, rated only on a 4-point scale (with 4-button response pad inside the MRI scanner), but they did show a trend towards racial bias in empathy consistent with the fMRI results.

It is still an open question, how racial differences cause changes in neural empathic responses. It may be that we are innately tuned to the perception of people who are “like us” [85]. Alternately, neural empathic responses to other races or groups may change with familiarity. We are generally more familiar with people of our own race than other races, which may facilitate the recognition of facial expressions and emotions [86], although in the current study, all faces displayed neutral expressions and so results cannot simply be due to differences in the perception of facial expressions across races. In a recent fMRI study, Azevedo et al. [30], examined empathic neural responses as participants observed same-race and other-race hands, as well as totally unfamiliar violet hands, receiving painful touch. Even though participants showed stronger activation in response to own race hands compared with the other hands in pain, they also showed increased activation in medial cingulate cortex and greater autonomic responses to other-race hands compared with the completely unfamiliar violet hand. Further studies are needed, however, to elucidate the role of familiarity in racial biases in neural empathy.

In summary, here we have shown a racial bias in neural empathic responses to pain in the left insula cortex (and similar trends in the anterior cingulate and somatosensory areas), confirming findings from a number of previous studies regarding racial biases in affective-motivational aspects of empathy [29]–[34]. Furthermore, we found that this racial bias persists and is not influenced by in-group bias in a minimal group context, even though participants clearly showed implicit and explicit identification with their minimal in-group rather than their racial group behaviourally. These results are consistent with an early and automatic brain response to observed pain that is modulated by race, and less influenced by meaningless or minimal group association. Importantly, behavioural measures in our study suggest that despite this racial bias in early neural responses, racial biases are not always reflected in our ultimate behaviour.
